# Combined internal neurolysis and targeted proximal trigeminal root glycerol delivery for refractory multiple sclerosis–related trigeminal neuralgia: a case report

**DOI:** 10.1186/s41016-026-00433-x

**Published:** 2026-05-14

**Authors:** Thomas Patrick Short, Chandrasekaran Kaliaperumal

**Affiliations:** Department of Neurosurgery, Royal Infirmary of Edinburgh, Edinburgh, UK

**Keywords:** Multiple sclerosis, Trigeminal neuralgia, Internal neurolysis, Glycerol injection, Salvage surgery

## Abstract

**Background:**

Trigeminal neuralgia associated with multiple sclerosis (MS-TN) is frequently refractory to medical and procedural therapy, particularly in the absence of neurovascular compression. Surgical options are limited when microvascular decompression is not appropriate. We report a salvage surgical approach combining internal neurolysis and targeted proximal trigeminal root glycerol injection for refractory MS-related trigeminal neuralgia.

**Case presentation:**

A 50-year-old woman with relapsing–remitting multiple sclerosis presented with medically refractory right-sided trigeminal neuralgia predominantly involving the V2/V3 distributions. She had previously failed optimisation of pharmacotherapy, two percutaneous retrogasserian glycerol rhizotomies, and Gamma Knife radiosurgery performed within the preceding year. Posterior fossa exploration via a retrosigmoid approach demonstrated no neurovascular conflict. Internal neurolysis (nerve combing) was therefore performed and supplemented with targeted glycerol delivery to the proximal cisternal segment of the trigeminal root adjacent to the radiologically relevant demyelinating lesion. Postoperatively, the patient experienced immediate improvement in facial pain with preservation of trigeminal sensation and no new neurological deficits. Her early postoperative course was complicated by a wound infection requiring surgical washout and antibiotic therapy. At 43-month follow-up, she remained free of ipsilateral trigeminal neuralgia with substantially reduced medication requirements. She later developed contralateral facial pain controlled with low-dose carbamazepine without recurrence on the operated side.

**Conclusion:**

This report describes, to our knowledge, the first published case of combined internal neurolysis and targeted proximal trigeminal root glycerol injection for refractory multiple sclerosis–related trigeminal neuralgia. In this highly selected case, durable ipsilateral pain control was observed. However, causal interpretation is limited by the single-case design, the combined nature of the intervention, and the potential delayed effects of prior radiosurgery. This approach should therefore be regarded as hypothesis-generating rather than practice-defining, but may merit further study as a salvage strategy in selected patients without neurovascular compression after failure of medical, percutaneous, and radiosurgical treatments.

## Background

Trigeminal neuralgia associated with multiple sclerosis (MS-TN) is an uncommon but debilitating condition affecting a minority of patients with demyelinating disease [1, 2]. Compared with idiopathic trigeminal neuralgia, MS-TN is often associated with more severe symptoms, higher rates of treatment failure, and less durable pain control [1, 3, 4]. Demyelination at or near the trigeminal root entry zone may promote ectopic impulse generation and ephaptic transmission rather than primary neurovascular compression [1, 5–7].

Management remains challenging. Pharmacotherapy is first-line, yet many patients develop medically refractory pain [1, 8]. Percutaneous procedures and stereotactic radiosurgery may provide temporary relief, but recurrence rates are higher in MS-TN [4, 9, 10]. In the absence of a compressive vascular target, microvascular decompression is frequently not feasible or effective, limiting surgical options [3, 5].

We present a case of refractory MS-TN without neurovascular compression treated with combined internal neurolysis and targeted proximal trigeminal root glycerol injection as a salvage strategy after failure of medical, percutaneous, and radiosurgical therapies. Evidence to guide further operative management in this setting remains limited. Internal neurolysis has been described in trigeminal neuralgia without neurovascular conflict, but evidence in MS-associated disease remains sparse, and the role of adjunct targeted chemical neurolysis at the proximal trigeminal root has not been defined within the broader spectrum of ablative or root-level salvage procedures [11–14]. To our knowledge, this represents the first published report of this combined technique in trigeminal neuralgia secondary to multiple sclerosis.

## Case presentation

A 50-year-old woman with relapsing–remitting multiple sclerosis presented with severe, medically refractory right-sided trigeminal neuralgia. Her neurological history was notable for optic neuritis with approximately 80% visual loss, and she was registered blind. She also had a history of migraine.

The trigeminal neuralgia manifested as paroxysmal, electric shock-like pain predominantly involving the maxillary and mandibular (V2/V3) distributions, resulting in substantial impairment of daily activities. The pain was provoked by routine activities including speaking, eating, and facial contact. Preoperatively, the patient’s pain severity was consistent with a Barrow Neurological Institute (BNI) pain intensity score of V.

**Fig. 1:**
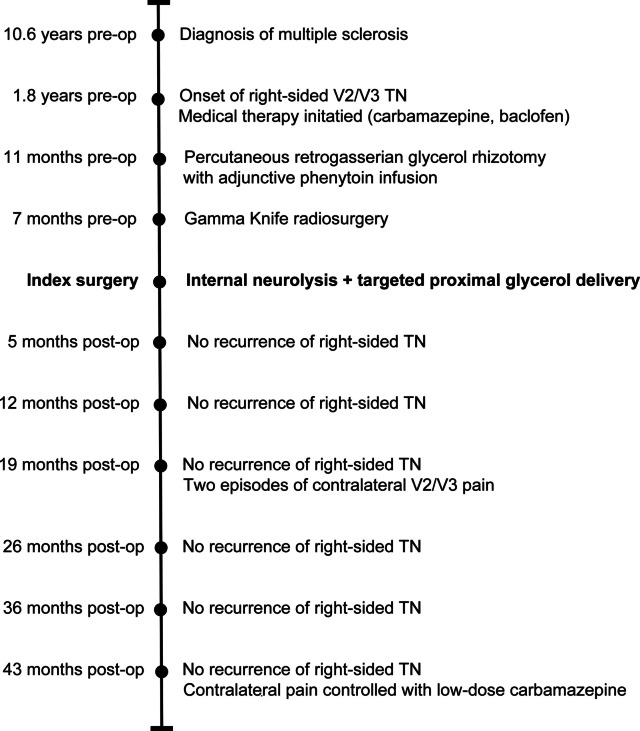
Clinical timeline of symptom progression, prior interventions, index surgery, and postoperative follow-up. Timeline illustrating the diagnosis of multiple sclerosis, onset of right-sided trigeminal neuralgia, prior medical and procedural treatments, index surgery consisting of internal neurolysis with targeted proximal glycerol delivery, and longitudinal follow-up. The patient remained free of recurrence of right-sided trigeminal neuralgia through 43 months following surgery, although intermittent contralateral facial pain later developed and was controlled medically

The chronological sequence of her symptoms and prior treatments is summarised in Fig. 1.

She required ongoing anticonvulsant therapy including carbamazepine and baclofen but continued to experience breakthrough pain despite medication escalation, prompting referral for neurosurgical assessment.

### Prior interventions

Prior to referral for posterior fossa exploration, the patient had undergone multiple procedural interventions for right-sided trigeminal neuralgia. She initially underwent percutaneous retrogasserian glycerol rhizotomy, which produced no meaningful benefit. A repeat glycerol rhizotomy was performed in July 2021 under general anaesthesia with fluoroscopic guidance, again without durable pain relief. Given ongoing medically refractory symptoms, she subsequently underwent Gamma Knife radiosurgery in December 2021. Despite this, she continued to experience recurrent paroxysmal facial pain requiring ongoing anticonvulsant therapy, and no clear or sustained clinical improvement was documented prior to consideration of posterior fossa exploration.

### Imaging and operative decision-making

**Fig. 2:**
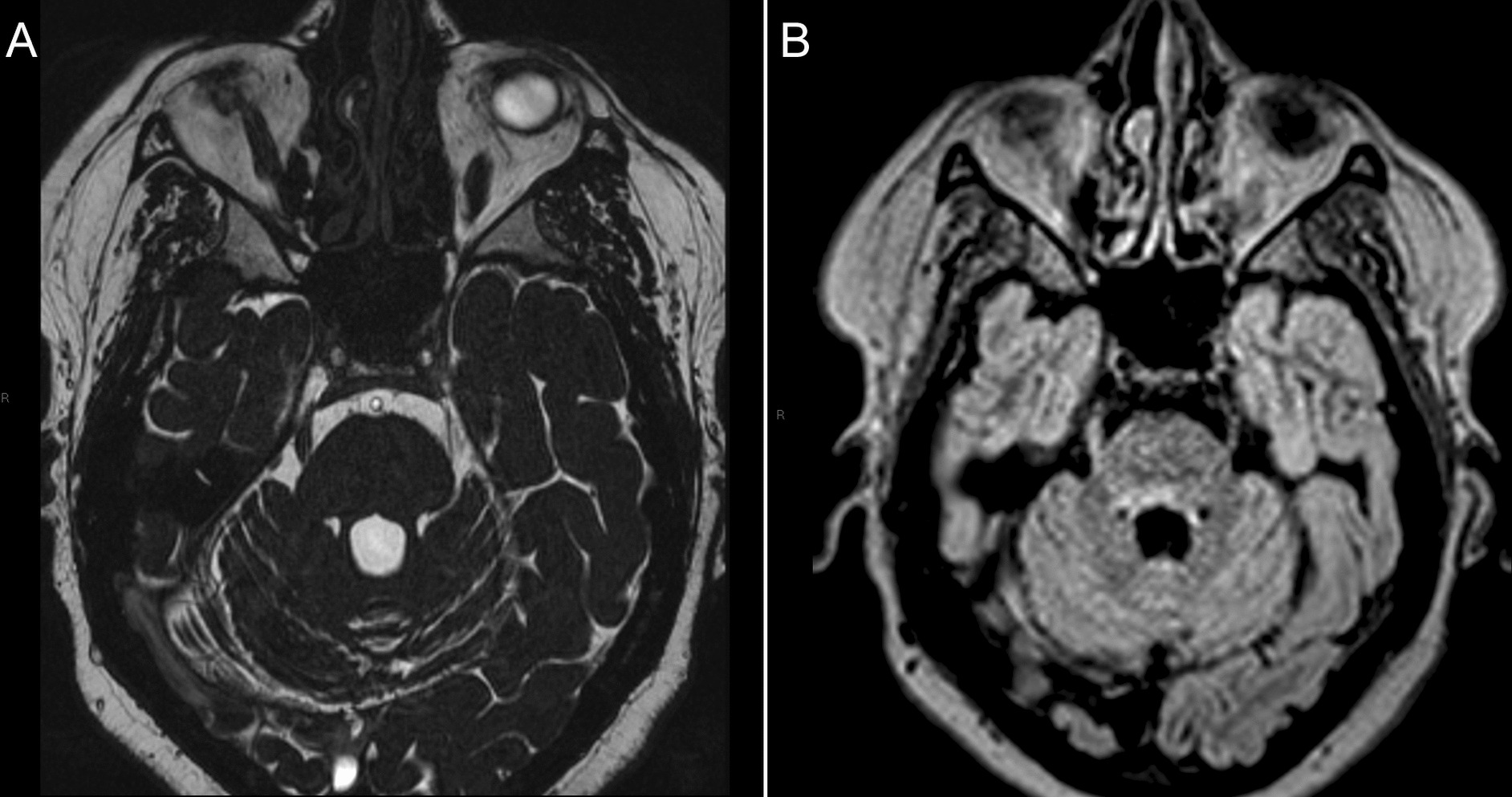
**A** Axial CISS sequence demonstrating the cisternal segment of the right trigeminal nerve without evidence of neurovascular compression. **B** Axial FLAIR image demonstrating hyperintense demyelinating signal within the brainstem extending toward the trigeminal root entry zone

Magnetic resonance imaging of the brain demonstrated multiple areas of demyelinating signal abnormality consistent with the patient’s known diagnosis of multiple sclerosis, with prominent involvement of the brainstem extending toward the trigeminal root entry zones bilaterally. Dedicated cranial nerve sequences did not demonstrate convincing neurovascular conflict involving the right trigeminal nerve. Comparison with prior imaging showed no significant interval change in the brainstem lesion adjacent to the trigeminal root entry zone (Fig. 2A and B).

Given the absence of a surgically meaningful vascular compression target and the failure of prior medical, percutaneous, and radiosurgical interventions, further escalation of conventional therapy was considered unlikely to provide durable benefit. Alternative salvage options were discussed in a multidisciplinary setting involving neurosurgery, pain, neurology, clinical psychology, and neuroradiology teams.

The operative rationale was based on the lack of a decompressive target and the radiological proximity of a stable demyelinating brainstem lesion to the trigeminal root entry zone. Posterior fossa exploration with trigeminal internal neurolysis was therefore offered. Adjunct targeted glycerol injection to the proximal trigeminal root was planned to address a more proximal anatomical segment than that treated by the patient’s prior retrogasserian glycerol procedures.

The risks of surgery, including persistent pain, facial sensory disturbance, and general operative complications, were discussed in detail, and written informed consent was obtained.

### Surgical technique

The patient was positioned in the park-bench right lateral position. Following induction of general anaesthesia, a lumbar puncture was performed with removal of 20 mL of cerebrospinal fluid to facilitate cerebellar relaxation. A right retrosigmoid craniotomy was performed using standard microsurgical technique.

After dural opening under the operating microscope, dense arachnoidal adhesions were encountered surrounding the trigeminal nerve, which appeared displaced superiorly. Dandy’s vein was noted to traverse the nerve without evidence of compression. No neurovascular conflict involving the trigeminal nerve was identified.

The trigeminal nerve was carefully exposed and internal neurolysis (nerve combing) was performed using standard microsurgical technique as previously described [11, 12]. Fascicular separation was carried out using Rhoton microinstruments, including a straight dissector and a 45-degree 3—mm ball probe. Approximately 8–10 fascicular cleavage planes were developed across the full width of the trigeminal nerve, extending to within approximately 2 mm of the dorsal root entry zone. This was performed as controlled interfascicular separation without intentional rhizotomy or fascicular sectioning.

Following completion of internal neurolysis, 0.3 mL of 100% anhydrous glycerol (sourced via NHS Lothian pharmacy) was delivered using a 1-mL syringe fitted with a 30G 1/2-inch Sol-M needle into a confined perineural plane along the surface of the trigeminal root (within the perineurial layer) at the inferior third of the proximal cisternal segment, anterior to the dorsal root entry zone, targeting the segment anatomically adjacent to the radiologically relevant demyelinating lesion. The selected volume was based pragmatically on volumes commonly used in percutaneous glycerol procedures, while recognising the differing anatomical target in this case [9, 14]. Care was taken to confine delivery to the intended nerve segment and avoid free spillage into the surrounding cerebrospinal fluid space. No intraoperative neurophysiological monitoring was used.

Haemostasis was secured and the dura was closed in a watertight fashion. Standard layered wound closure was performed.

Postoperatively, there was no clinically significant new facial numbness or dysaesthesia. Corneal reflexes were intact, light-touch sensation in the V1–V3 distributions was preserved on bedside examination, and there was no clinical evidence of masticatory weakness.

Her early postoperative course was complicated by a superficial wound infection requiring surgical washout on postoperative day 11 and antibiotic therapy. There was no evidence of intracranial infection or trigeminal-specific neurotoxic sequelae. The patient was also receiving immunotherapy for multiple sclerosis, which may have been a contributory factor.

At 5-month follow-up, she reported significant improvement in trigeminal neuralgia with only occasional residual stabbing pain.

At 12-month follow-up, she reported no recurrence of right-sided trigeminal neuralgia. Her postoperative pain status was consistent with a BNI pain intensity score of I. Analgesic and anticonvulsant requirements had been substantially reduced and she was in the process of weaning carbamazepine.

At 19- and 26-month follow-up, she continued to remain free of right-sided trigeminal neuralgia. She developed intermittent contralateral facial pain involving the left V2/V3 distributions, controlled with low-dose carbamazepine (100 mg twice daily). There was no clinical evidence of recurrence on the operated side.

She therefore remains pain-free on the operated side 43 months post-operatively, with an overall postoperative course consistent with improvement from a preoperative BNI pain intensity score of V to a postoperative score of I.

## Discussion

### Clinical significance of the present case

This case describes durable ipsilateral pain freedom following combined internal neurolysis and targeted proximal trigeminal root glycerol injection in a patient with refractory multiple sclerosis–related trigeminal neuralgia (MS-TN) who had previously failed multiple medical and procedural therapies. To our knowledge, this represents the first published report of this combined approach in MS-associated trigeminal neuralgia. Sustained relief was observed on the operated side despite the absence of neurovascular compression and the presence of demyelinating disease involving the trigeminal root entry zone. This was accompanied by improvement from a preoperative BNI pain intensity score of V to a postoperative score of I, without clinically evident corneal dysfunction, facial sensory loss, or masticatory weakness.

MS-TN is mechanistically distinct from idiopathic trigeminal neuralgia, with demyelination at or near the trigeminal root entry zone thought to promote ectopic impulse generation and ephaptic transmission [5–7]. As a result, conventional operative strategies—particularly microvascular decompression—may be less suitable or less effective in this subgroup when a clear compressive vascular target is absent [3, 5].

### Mechanistic rationale for the combined approach

Internal neurolysis aims to mechanically disrupt abnormal cross-talk between demyelinated trigeminal fascicles, while focal glycerol delivery may provide adjunctive chemical neurolysis with preferential effects on nociceptive fibres, consistent with longstanding lesioning principles in trigeminal pain surgery [14, 15]. Internal neurolysis has previously been described for trigeminal neuralgia in the absence of neurovascular conflict, including contemporary technical series, early UK experience, and reports from Asian neurosurgical centres, reflecting growing international interest in root-level strategies [11, 12, 16–18].

In the present case, adjunct targeted proximal trigeminal root glycerol injection was used to address a more proximal anatomical segment than that treated by the patient’s prior retrogasserian glycerol procedures. The rationale was to target the cisternal segment of the trigeminal root adjacent to the radiologically relevant brainstem lesion, rather than the ganglion or Meckel’s cave region approached during percutaneous treatment. In this context, the intended target was the proximal cisternal segment of the trigeminal root itself rather than the surrounding cerebrospinal fluid space.

Internal neurolysis and related trigeminal nerve salvage strategies have been explored across multiple centres, including recent series from Chinese neurosurgical groups, reflecting ongoing international interest in root-level operative approaches in the absence of a clear decompressive target [16, 17].

Although these interventions may have acted through partially distinct mechanisms, their relative contributions cannot be determined from a single case.

### Position within current salvage treatment options

Management of refractory MS-TN in the absence of neurovascular compression remains challenging. Available salvage options include repeat percutaneous procedures, stereotactic radiosurgery, internal neurolysis, and more destructive open procedures such as partial sensory rhizotomy or microsurgical rhizotomy [4, 9, 10, 13, 14, 19]. Retrogasserian glycerol rhizotomy may provide transient benefit, but recurrence is common in MS-TN, while radiosurgery may offer delayed but sometimes less durable pain control in demyelinating disease [4, 9, 10]. Open trigeminal root lesioning procedures remain part of the broader salvage surgical spectrum in selected refractory cases, although they may carry a greater risk of postoperative sensory morbidity [13, 14, 19].

Internal neurolysis provides an alternative open surgical strategy in patients without a decompressive target [11, 12]. In contrast to retrogasserian glycerol procedures, the present approach attempted to address the proximal trigeminal root directly through a retrosigmoid exposure. However, this report does not establish superiority over internal neurolysis alone, nor does it allow determination of whether the observed outcome was attributable to internal neurolysis, focal glycerol delivery, prior radiosurgical treatment, or a combination of these factors.

### Clinical considerations and patient selection

This case underscores several practical considerations in the management of refractory MS-TN. In patients without neurovascular compression who have failed medical, percutaneous, and radiosurgical treatments, posterior fossa exploration with internal neurolysis may be considered in highly selected cases. Any adjunctive proximal trigeminal root intervention should be regarded as experimental and reserved for carefully counselled patients in whom more established options have been exhausted.

Careful patient selection and preoperative counselling are therefore essential. This strategy should not be considered a first-line intervention but rather a highly selective salvage approach. The risks of surgery—including facial sensory disturbance, dysaesthesia, anaesthesia dolorosa, recurrence, and general operative complications—must be balanced against the morbidity of severe, medically refractory facial pain [11, 13, 14, 19]. Preservation of trigeminal sensation and avoidance of disabling numbness remain key goals in this population.

Long-term follow-up is also particularly important in MS-TN, as disease progression may result in bilateral symptoms, recurrence, or evolution of pain phenotype independent of the treated side. In the present case, the patient later developed contralateral facial pain without recurrence on the operated side, underscoring the dynamic and multifocal nature of trigeminal involvement in multiple sclerosis.

### Limitations

This report has several important limitations. First, it describes a single patient and therefore cannot establish efficacy, reproducibility, comparative benefit, or procedural safety. Second, the intervention was combined in nature, and it is not possible to determine whether the observed outcome was attributable predominantly to internal neurolysis, targeted proximal glycerol injection, or their interaction.

A further major limitation is the potential confounding effect of prior Gamma Knife radiosurgery. As the patient underwent Gamma Knife treatment within the preceding year, delayed or cumulative radiosurgical effects cannot be excluded. Therefore, the observed long-term pain relief cannot be causally attributed to the open procedure, and the relative contribution of prior radiosurgery versus the combined surgical intervention remains uncertain.

## Conclusion

This report describes, to our knowledge, the first published case of combined internal neurolysis and targeted proximal trigeminal root glycerol injection for refractory multiple sclerosis–related trigeminal neuralgia. In this highly selected case, durable ipsilateral pain control was observed. However, causal interpretation is limited by the single-case design, the combined nature of the intervention, and the potential delayed effects of prior radiosurgery. This approach should therefore be regarded as hypothesis-generating rather than practice-defining, but may merit further study as a salvage strategy in selected patients without neurovascular compression after failure of medical, percutaneous, and radiosurgical treatments.

## Data Availability

All data generated or analysed during this study are included in this published article.

## Abbreviations

MS: Multiple sclerosis
TN: Trigeminal neuralgia
MS-TN: Multiple sclerosis–related trigeminal neuralgia
V2: Maxillary division of the trigeminal nerve
V3: Mandibular division of the trigeminal nerve
